# Effects of acupoint massage combined with relaxation therapy on patients with postoperative fatigue syndrome after lumbar surgery

**DOI:** 10.1097/MD.0000000000025849

**Published:** 2021-05-14

**Authors:** Qiuhui Zheng, Rongyun Wang, Yanan Shi, Qiuhua Sun

**Affiliations:** a Hangzhou TCM Hospital Affiliated to Zhejiang Chinese Medical University; b School of Nursing, Zhejiang Chinese Medical University, Hangzhou, China.

**Keywords:** acupoint massage, lumbar disc herniation, postoperative fatigue syndrome, relaxation therapy

## Abstract

**Background:**

: Lumbar disc herniation (LDH) is a common disease in orthopedics. Surgery is shown to provide significant faster relief of pain compared to conservative therapy. However, due to the influence of surgical trauma, anesthesia and other perioperative stress factors, patients may have complications. Among them, postoperative fatigue syndrome (POFS) is a common complication. Traditional Chinese medicine or integrated traditional Chinese and Western medicine have been proved to be effective in improving postoperative fatigue.

**Methods:**

: This study is a randomized controlled trial. One hundred eighty Chinese patients with POFS of LDH will be randomly divided into control group, experimental group 1, experimental group 2 and experimental group 3 according to the ratio of 1:1:1:1. The patients in the control group will be treated with conventional treatment after operation, the patients in the experimental group 1 will be treated with acupoint massage, the patients in the experimental group 2 will be treated with relaxation therapy, and the patients in the experimental group 3 will be treated with acupoint massage combined with relaxation therapy. The whole treatment will last for 5 days. The main outcome measures will be fatigue visual analogue scale and identity-consequence fatigue scale, and the secondary outcome measures will be hospital anxiety and depression scale.

**Discussion:**

: This study is to observe the effects of acupoint massage comblined with relaxation therapy on reducing postoperative fatigue of lumbar disc herniation surgical patients.

**Trial registration:**

: Chinese Clinical Trial Registry (http://www.chictr.org.cn/edit.aspx?pid=123978&htm=4), No. ChiCTR2100044788. Registered on March 27, 2021.

## 1 Introduction

Lumbar disc herniation consists of displacement of the content of the intervertebral disc (the pulpous nucleus) through its external membrane (the fibrous ring), generally in its posterolateral region. Lumbar disc herniation (LDH) is a displacement of the content of the intervertebral disc (the pulpous nucleus) through its external membrane (the fibrous ring), generally in its posterolateral region, represented clinically by pain in low back, sciatica, intermittent claudication and cauda equina syndrome, which can affect limbs function and the quality of life.^[1]^ In recent years, the prevalence of LDH ranges from 3.7% to 5.1% in western developed countries,^[2]^ from 1% to 2% in the United States,^[3]^ and from 1% to 3% in Finland and Italy.^[4]^ According to the 2016 Chinese surgical Yearbook, the incidence of LDH has reached 2.9% in China, with as many as 300 million people affected daily life and work due to low back and leg pain caused by LDH.^[5]^ The current treatments for LDH mainly include pharmacotherapy, physical therapy (including traction therapy, Tuina, massage), surgical therapy, etc.^[6,7]^ Despite the great advances in surgical treatment of LHD with the advancement and development of medical science, the trauma, anesthesia of surgery, as well as the effects of other stressors in the perioperative period, still cause some complicationts to patients. Postoperative fatigue syndrome (POFS) is a common complication after surgery, which refers to fatigue burnout syndrome that occurs in surgical patients to a variable degree and for a variable length of time during the postoperative recovery period, and is dominated by poor concentration, fatigue, anxiety, depression, sleep disturbance, and decreased appetite.^[8]^ POFS will not only affect postoperative rehabilitation and cause distress for patients, but may also lead to secondary complications, resulting in prolonged hospitalization and decreased functional recovery, affecting patients’ return to society and imposing harms and burdens on families and society,^[9]^ and its incidence in postoperative patients is approximately 34% to 87%.^[10–12]^

There are drug therapy and non drug therapy for POFS. Drug therapy should take into account the patient's physical and other medication conditions, and its safety needs to be further proved. Compared with drug therapy, non drug therapy is safer. Relaxation therapy, mindfulness decompression and other non drug treatment have made some progress.^[13,14]^ Acupuncture and moxibustion, auricular point pressing and acupoint massage have been used in previous studies. Our study is to compare the efficacy of acupoint massage, relaxation therapy and acupoint massage combined with relaxation therapy on patients with postoperative fatigue after LHD surgery.

## 2 Methods

### 2.1 Registration

**Figure 1:**
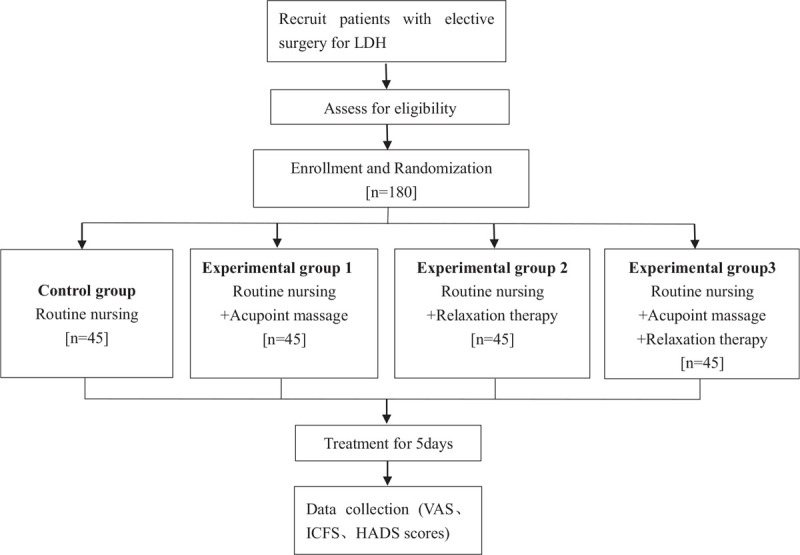
Study flowchart.

This study was registered in China clinical trial registry before recruitment (http://www.chictr.org.cn/showproj.aspx?proj=123978 No= ChiCTR2100044788). We will abide by the principles of the Helsinki Declaration (2004 version) during the trial. This study has been approved by the ethics committee of Zhejiang Provincial Hospital of traditional Chinese medicine and 903 Hospital of the Chinese People's Liberation Army joint logistics support force. Figure 1 shows a diagram with the different phases of the study.

### 2.2 Recruitment

This study will be recruited in Zhejiang Provincial Hospital of Traditional Chinese Medicine. Participants will be recruited through the recommendation of orthopedic surgeons. Before the enrollment, participants will be informed with detailed information about the clinical study, including its purpose, processing, scheduling, and possible risks and benefits. Only those who agree to sign the informed consent and voluntary participation in the trial will be included in the study.

### 2.3 Inclusion criteria

Patients undergoing elective surgery for LDH; Preoperative Visual Analogue Scales (VAS) score <3 points, subjects diagnosed with POFS, who are over 18 years old, normal cognitive ability; length of stay ≥7 days All Included patients had signed informed consent and voluntarily cooperated with this study signed informed consent and voluntarily cooperated with this study.

### 2.4 Exclusion criteria

Patients with other serious diseases and severe fatigue before operation (Preoperative VAS score ≥3 points). Patients with unconsciousness or severe communication disorder and unstable vital signs will also be excluded.

### 2.5 Sample size estimation

Pass software was used to estimate the sample size. According to the results of the preliminary study, the identity-consequence fatigue scale (ICFS) score was -2.80 ± 12.82 in the control group, -33.63 ± 10.01 in the acupoint massage group, -12.95 ± 8.41 in the relaxation therapy group, and −40.90 ± 7.53 in the acupoint massage combined with relaxation therapy group. Under the condition of α = 0.05 and test efficiency 1-β = 0.8, 37 cases in each group were calculated. Considering the loss of follow-up, shedding and other factors, the sample size was expanded by 20%, and finally each group was determined. There will be 45 cases in each group and 180 cases in total.

### 2.6 Randomization

SPSS software was used to generate 180 random numbers, and the rank of the 180 random numbers was 1 to 180. The random numbers were selected according to the order of inclusion. The research objects with the corresponding rank of 1 to 45, 46 to 90, 91 to 135 and 136 to 180 were divided into control group, experimental group 1, experimental group 2, and experimental group 3.

### 2.7 Interventions

The patients with selective LDH operation in two hospitals will be selected. The fatigue will be assessed by VAS 1 day before operation, and the patients with score less than 3 pass the initial screening; the patients who passed the initial screening will be reassessed 1 day after operation (no more than 24 hours), and the patients with VAS score ≥3 and meeting the POFS diagnostic criteria, inclusion and exclusion criteria will be finally included.

The subjects will be divided into control group, experimental group 1 (acupoint massage), experimental group 2 (relaxation therapy) and experimental group 3 (acupoint massage combined with relaxation therapy) according to the ratio of 1:1:1:1. The patients in the control group will only receive routine perioperative nursing; the patients in experimental group 1, experimental group 2 and experimental group 3 will receive the same routine perioperative nursing as the control group, and the patients in experimental group 1 will also receive acupoint massage, once a day, 30 minutes each time; experimental group 2 will also receive relaxation therapy, once a day, 20 minutes each time; experimental group 3 will also receive acupoint massage combined with relaxation therapy (i.e., acupoint massage first, and then relaxation therapy, once a day, 50 minutes each time), the intervention time of 4 groups were from the 1st day to the 5th day after operation.

The standard treatment procedure of acupoint massage are as follows: The patient will be in the supine position. The operator will selects acupoints Baihui, Fengchi, Neiguan, Shenmen, Zusanli, Sanyinjiao, Chengshan and Kunlun, use pressing method and kneading method. Bilateral acupoints (Fengchi, Neiguan, Zusanli, Sanyinjiao, Chengshan and Kunlun) will be massaged with both hands at the same time. Each acupoint will be massaged for 3 to 4 minutes, 120 to 160 times/min. Massage intensity from light to heavy, from heavy to light.

The standard treatment procedure of relaxation therapy are as follows: Let the patient take the supine position to make him feel comfortable, tell the patient to pay attention to breathing, and do corresponding actions according to the prompt sound. After each action is tense for 10 seconds, gradually relax, and the relaxation action lasts for 10 seconds. Feel the difference between muscle tension and relaxation in all parts of the body. The actions are as follows: clench fist, shrug up, shrug back, head down, clench teeth, and stretch Open your eyes wide, frown hard, close your eyes tightly, tighten your abdomen, tighten your hips, straighten your legs and lift them up, with the back of your feet up, the back of your feet down, the toes up and the toes down.

## 3 Data collection and management

### 3.1 Evaluation

#### 3.1.1 Primary outcome

The primary outcome will be postoperative fatigue. VAS and ICFS will be used to assess the degree of fatigue before and after intervention. VAS divides the fatigue degree into 1 to 10 points, and the patients select the number according to their own fatigue degree.^[15]^ ICFE is used to measure perioperative fatigue, which is composed of 5 dimensions and 31 items, and its score ranges from - 88 to 67.^[16]^ The higher the scores of VAS and ICFS, the more severe the fatigue.

#### 3.1.2 Secondary outcome measurements include anxiety and depression

Hospital anxiety and depression scale will be used to assess the degree of anxiety and depression.^[17]^ Hospital anxiety and depression scale consists of 2 subscales: anxiety and depression, each with 7 items. The highest score of each subscale is 21. Any subscale score of 8 or higher is considered to be anxious or depressive. Anxiety and depression are also the main symptoms of POFS. The higher the scale score is, the higher the postoperative fatigue level is.

### 3.2 Statistical analysis

The researchers will collect the personal information of the participants and recorded the scoring results. And it will be updated in China clinical trial registry before and during the trial. The measurement data will be described by mean ± standard deviation, and the count data will be described by frequency and percentage. The value of α 0.05, and the value of *P* is bilateral probability. When *P* < .05, there will be considered to be statistically significant.

## 4 Discussion

Lumbar disc herniation is the most common diagnosis among the degenerative abnormalities of the lumbar spine (affecting 2% to 3% of the population), and is the principal cause of spinal surgery among the adult population. Because the operation has certain trauma, and most of the patients with LDH are middle-aged and elderly patients, it is easy to lead to postoperative complications, among which POFS is a common complication of LDH operation, it will prolong the time of patients in bed after operation, can not start functional exercise as soon as possible.^[18]^ In fact, most of the evidence related to the postoperative period was obtained during the first 7 days after surgery, if there is no early intervention, it will last longer, even 12 months after surgery.^[19]^ It may also cause secondary complications, prolong the length of hospital stay, and affect the patients’ return to society.^[20]^ Acupoint massage stimulates acupoints by massage, so as to adjusting activity of Qi, activate meridians, promote blood circulation, strengthen metabolism, enhance immunity and promote sleep, so as to achieve the balance of Yin and Yang and relieve fatigue. Wolpe's interactive inhibition theory thinks that relaxation therapy can inhibit anxiety, tension, depression and other negative emotions through muscle relaxation.^[21]^ On the one hand, the relaxation therapy can distract the attention of patients and reduce the muscle tension of patients. At the same time, the nerve electrical activity in frontal lobe brain area changes, the excitability of parasympathetic nerve increases and the excitability of sympathetic nerve decreases, so as to improve the tolerance of external stressors and relieve the fatigue caused by muscle tension.^[22]^ The mechanism of acupoint massage and relaxation therapy is different. If combined with the 2 techniques, it can not only dredge the whole body, regulate the function of viscera, but also balance the activities of sympathetic nerve and parasympathetic nerve, and can resist the problems of functional disorder and mental fatigue caused by surgical stress in patients with POFS.

In view of the high incidence of LDH and the possible complications caused by surgical treatment, we try to carry out a prospective trial to find an early intervention method for postoperative fatigue syndrome of LDH, so as to provide practical basis for clinical practice, and hope to promote the postoperative rehabilitation of patients and reduce complications Appendix A.

### 4.1 Trial status

The protocol's version number and date are V1.0 and May 30, 2019. The first participants were recruited in February 1, 2021 and will be finished approximately in September 30, 2021. The recruitment is currently open. The data will be updated in the Chinese Clinical Trial Registry.

## Author contributions

**Conceptualization:** Qiuhui Zheng, Qiuhua Sun.

**Methodology:** Rongyun Wang.

**Resources:** Qiuhui Zheng, Rongyun Wang, Yanan Shi.

**Supervision:** Qiuhua Sun.

**Writing – original draft:** Qiuhui Zheng.

**Writing – review & editing:** Rongyun Wang, Qiuhua Sun.

## Supplementary Material

Supplemental Digital Content
