# Supplementary material for: Effects of acupoint massage combined with relaxation therapy on patients with postoperative fatigue syndrome after lumbar surgery
Source: Medicine (Baltimore). 2021 May 14;100(19):e25849. doi: 10.1097/MD.0000000000025849 (PMC8133127; doi:10.1097/MD.0000000000025849)
Supplement: Supplemental Digital Content [file medi-100-e25849-s001.doc]

**Appendices 1 [Informed](../../../../D:/%25E6%259C%2589%25E9%2581%2593/Dict/8.9.6.0/resultui/html/index.html#/javascript:;) [Consent](../../../../D:/%25E6%259C%2589%25E9%2581%2593/Dict/8.9.6.0/resultui/html/index.html#/javascript:;)**

穴位按摩联合放松训练对腰椎间盘突出术后疲劳综合征的干预研究

知情同意书·知情告知页

**版本号：V1.0**

**版本日期: 2019.5.30**

亲爱的患者：

经评估已经确定您患有术后疲劳综合征。

我们将邀请您参加一项《穴位按摩联合放松训练对腰椎突出术后疲劳综合征的干预研究》的试验性研究，以观察该干预方案对于术后疲劳综合征的疗效和安全性。

在您决定是否参加这项研究之前，请尽可能仔细阅读以下内容，它可以帮助您了解该项研究以及为何要进行这项研究，研究的程序和期限，参加研究后可能给您带来的益处、风险和不适。如果您愿意，您也可以和您的亲属、朋友一起讨论，或者请研究者给予解释，帮助您做出决定。

研究介绍

**一、研究背景和研究目的**

国外相关研究文献显示，腹部手术的患者更易发生术后疲劳综合征，Lin等调查发现肝移植术后疲劳综合征的发生率高达87%。Yu等研究发现胃肠道手术中重度以上术后疲劳综合征的发生率为41.3%。Wikman等研究报道30%的食管癌患者在术后6个月仍然感到疲劳。经临床观察显示，术后疲劳综合征是腰椎术后常见的并发症，其主要症状是感觉乏力、疲劳、嗜睡，会影响术后的早期康复，甚至导致二级并发症。

目前国内外针对术后疲劳综合征患者治疗干预方法主要有药物治疗、非药物干预等方法。但由于腰椎疾病的患者多以中老年为主，多数患者可能还兼有其他合并症，在对术后疲劳综合征患者用药的时候，也需兼顾其他系统的疾病，所以针对术后疲劳综合征的药物治疗效果尚难确定。因此，近年来非药物治疗越来越受到重视，具有安全、经济、有效的特点，从而易于被患者接受，且有利于护患沟通，从而降低患者不良情绪，提高护理满意度。

该研究目的：为了帮助患者缓解腰椎术后疲劳症状，减少二级并发症的发生，促进患者术后康复，并为今后更有效地临床干预术后疲劳综合征提供方法和实践依据。。

伦理委员会已经审议此项研究是遵从赫尔辛基宣言原则，符合医疗道德的。

**二、哪些人不宜参加研究**

(1)合并肺心病、呼吸衰竭的患者；

(2)术前伴有严重疲劳者；

(3)术后生命体征不稳定的患者。

(4)意识不清或存在严重沟通障碍者。

**三、如果参加研究将需要做什么**

1. 在您入选研究前，您将接受以下评估以确定您是否可以参加研究：

研究者将询问、记录您的病史。

您需要做围手术期疲劳、睡眠、焦虑、抑郁等评估。

2. 若您已完成以上评估，将按以下步骤进行研究

研究开始将根据计算机提供的随机数字，决定您接受试验组或对照组方案。参加这项研究的患者分别有20%的可能性被分入这四个不同的组别。您和本项目研究者都无法事先知道和选择任何一种干预措施。治疗观察将持续5天。

①干预前：疲劳视觉模拟评分（VAS）、围手术期疲劳测评量表（ICFS）、医院焦虑抑郁量表（HADS）、评分；

②术后3天：VAS、ICFS、HADS评分；

③术后5天：VAS、ICFS、HADS评分。

3. 需要您配合的其他事项

术后将会定期进行干预和评估，对您的评估非常重要，因为研究者将判断您接受的研究措施是否真正起作用。

**四、参加研究可能的受益**

您和社会将可能从本项研究中受益。此种受益包括您的病情有可能获得改善，以及本项研究可能帮助开发出一种新干预方案，以用于患有相似病情的其他病人。

您将在研究期间获得良好的医疗服务，享受免费咨询、穴位按摩和放松训练。

**五、参加研究可能的不良反应、风险和不适、不方便**

放松训练的不良反应：肌肉紧张过程中过度用力导致肌肉拉伤。

尽管到目前为止没有发现该研究方法有任何不良反应，如果在研究中您出现任何不适，或病情发生新的变化，或任何意外情况，不管是否与干预方法有关，均应及时通知您的医生和护士，他/她将对此作出判断和医疗处理。

课题组将尽全力预防和治疗由于本研究可能带来的伤害。如果在临床研究中出现不良事件，科研项目所属政府管理部门及医院伦理委员会将会鉴定其是否与本研究有关。研究者将对与研究相关的损害提供治疗的费用及相应的经济补偿。

此外，（研究干预）可能出现无效的情况，以及因治疗无效或者因合并其他疾病等原因而导致病情继续发展。在研究期间，如果研究者发现本项研究所采取的（研究干预）措施无效，将会中止研究，改用其他可能有效的治疗措施。

**六、有关费用**

如果发生与研究相关的损害，研究者将支付您的医疗费用。如果因与研究相关的严重不良反应住院医疗，研究者还将提供适当的补偿，包括适当的营养费、误工的工资和奖金的补偿费。如果您同时合并其他疾病所需的治疗和检查，将不在免费的范围之内。

**七、个人信息是保密的吗？**

您的医疗记录（研究病历/CRF、化验单等）将完整地保存在医院，医生会将各种检查结果记录在您的病历上。研究者或课题组成员、伦理委员会和课题所属政府部门将被允许查阅您的医疗记录。任何有关本项研究结果的公开报告将不会披露您的个人身份。我们将在法律允许的范围内，尽一切努力保护您个人医疗资料的隐私。

除本研究以外，有可能在今后的其他研究中会再次利用您的医疗记录和检查结果。您现在也可以声明拒绝除本研究外的其他研究利用您的医疗记录和检查结果。

**八、怎样获得更多的信息？**

您可以在任何时间提出有关本项研究的任何问题。研究者将给您留下他/她的电话号码以便能回答您的问题。

如果您对参加研究有任何抱怨，请联系医院伦理委员会办公室。

如果在研究过程中有任何重要的新信息，可能影响您继续参加研究的意愿时，研究者将会及时通知您。

**九、可以自愿选择参加研究和中途退出研究**

是否参加研究完全取决于您的自愿。您可以拒绝参加此项研究，或在研究过程中的任何时间退出本研究，这都不会影响您和医护人员的关系，都不会影响对您的医疗或有其他方面利益的损失。

研究者出于对您的最大利益考虑，可能会随时中止您参加本项研究。

您可以不参加本项研究，或中途选择退出研究。

如果您因为任何原因从研究中退出，您可能被询问有关您干预过程中的情况。如果研究者认为需要，您也可能被要求进行实验室检查和体格检查，这对保护您的健康十分有利。

**十、现在该做什么？**

是否参加本项研究由您自己决定。您可以和您的家人或者朋友讨论后再做出决定。

在您做出参加研究的决定前，请尽可能向本研究研究者询问有关问题，直至您对本项研究完全理解。

感谢您阅读以上材料。

如果您决定参加本项研究，请告诉研究者，他/她会为您安排一切有关研究的事务。

请您保留这份资料。

知情同意书·同意签字页

临床研究项目名称：穴位按摩联合放松训练对腰椎间盘突出术后疲劳综合征的研究

临床研究开展单位：浙江省中医院、中国人民解放军联勤保障部队第903医院

伦理审查批件号： 伦理审查批件右上角

同意声明

我已经阅读了上述有关本研究的介绍，而且有机会就此项研究与医生讨论并提出问题。我提出的所有问题都得到了满意的答复。

我知道参加本研究可能产生的风险和受益。我知晓参加研究是自愿的，我确认已有充足时间对此进行考虑，而且明白：

Ⅰ我可以随时向研究者咨询更多的信息。

Ⅱ我可以随时退出本研究，而不会受到歧视或报复，医疗待遇与权益不会受到影响。

我同样清楚，如果我中途退出研究，特别是由于药物的原因使我退出研究时，我若将病情变化告诉医生，完成相应的体格检查和理化检查，这将对我本人和整个研究十分有利。

如果因病情变化我需要采取任何其他的药物治疗，我会在事先征求医生的意见，或在事后如实告诉医生。

我同意伦理委员会或申办者代表及研究质量监察人员查阅我的研究资料。

我同意□ 或拒绝□ 除本研究以外的其他研究利用我的医疗记录和病理检查标本。

我将获得一份经过签名并注明日期的知情同意书副本。

最后，我决定同意参加本项研究。

受试者签名： 　＿ ＿ ＿ ＿ 年 ＿ ＿ 月 ＿ ＿ 日

受试者联系电话： 手机号：

法定代理人签名（如有）： 日期： 年 月 日

我确认已向患者解释了本研究的详细情况，包括其权利以及可能的受益和风险，并给其一份签署过的知情同意书副本。

研究者签名： 日期：＿ ＿ ＿ ＿ 年 ＿ ＿ 月 ＿ ＿ 日

研究者工作电话： 手机号码：

**Appendices 1 Study protocol**

穴位按摩联合放松训练对腰椎间盘突出术后疲劳综合征的干预研究

**研究方案及计划**

**版本号：V1.0**

**版本日期: 2019.5.30**

**一、研究背景**

腰椎间盘突出症（lumbar disc herniation，LDH）是常见的骨科疾病，文献提示，目前LDH治疗方法主要有保守治疗、手术治疗等，随着医学科技的进步和发展，越来越多的患者进行手术治疗，这与手术技术的发展及患者对健康需求的改变有显著关系。然而由于患者个人因素，加之手术创伤、麻醉及围手术期其他应激因素的影响，患者术后易发生并发症。其中，术后疲劳综合征（postoperative fatigue syndrome，POFS）是患者LDH术后多见的一种并发症，是影响患者术后康复的危险因素之一。患者发生POFS不仅会影响术后康复，给患者带来痛苦，还会导致二级并发症，如感染、压疮、功能障碍等，导致住院时间延长、功能恢复下降，影响患者回归社会，给家庭和社会带来危害和负担。因此，由于POFS的易发性以及可能导致的危害性，腰椎POFS已越来越受到医学界重视，并逐渐成为外科手术后医护关注和研究的重点。探索腰椎POFS有效干预的方法对促进患者术后康复，提高生存质量及推动术后康复护理的学术进步具有重要意义。

因此，国内外学者越来越重视POFS相关研究，在治疗方面非药物干预具有优势，文献提示目前干预研究大多为单一干预方法，研究设计在规范性和科学性上尚存在问题，因此采用科学的方法规范设计，选择有效的干预方法具有很强的必要性。经查询和临床经验总结提示穴位按摩和放松训练对于缓解疲劳、焦虑、抑郁等有确切的作用，这两种方法具有操作方便、安全无创、经济易行、患者易接受的特点，因其作用效果及推广应用上的优势，易于被医护人员接受，且日益受到医护界的重视和关注。但通过文献研究提示，目前未见国内外关于穴位按摩联合放松训练对腰椎POFS干预的文献报道，鉴于目前国内外对腰椎POFS干预研究的现状和存在的问题以及开展进一步研究的必要性，在总结前人对相关研究成果和借鉴临床实践经验的基础上，根据POFS的发生机理，选择穴位按摩和放松训练的方法对腰椎POFS进行护理干预研究，探索腰椎POFS有效的干预方法，为临床提供干预治疗腰椎POFS提供实践依据，对促进腰椎术后的康复及提高生活质量具有重要意义。

**二、研究设计**

**（一）研究内容**

本研究采用随机对照研究研究方法。运用经文献查询、总结临床经验以及专家咨询的基础上形成的穴位按摩联合放松训练的干预方案，干预腰椎间盘突出症手术的术后疲劳综合征，观察患者的临床疗效、疲劳、抑郁、焦虑及睡眠情况等改变。评价穴位按摩以及放松训练在改善术后疲劳综合征方面的有效性，为后续的进一步研究奠定基础。

**（二）研究方案**

**1、研究对象和场所**

本研究以中国人民解放军联勤保障部队第903医院骨二科为研究场所。

**（1）纳入标准**

①疾病诊断符合2009年中华医学会编着的第1版《临床诊疗指南一骨科分册》中腰椎间盘突出症的诊断标准，并经腰椎CT或MRI证实。

②进行外科择期腰椎手术的患者。

③术前疲劳视觉模拟评分＜3分的患者。

④术后符合POFS诊断标准的患者。

⑤不合并有严重其他疾病。

⑥年龄≥18周岁。

⑦具有正常的认知能力。

⑧住院天数≥7天。

⑨签署知情同意书，自愿配合本研究。

**（2）排除标准**

①合并有严重内科疾病术前并伴有严重疲劳者（术前疲劳视觉模拟评分≥3分）。

②术后生命体征不稳定的患者。

③意识不清或存在严重沟通障碍者。

**（3）剔除标准和脱落病例标准**

①不符合纳入标准而被误纳入者。

②虽符合纳入标准而纳入后未按计划执行干预方案者。

③病情加重的患者。

④治疗过程中自动退出或发生意外情况不能坚持治疗者。

⑤实验过程中发生严重不良反应或特殊生理变化，不宜接受实验，并记录不良反应。

⑥资料不完善，影响有效性和安全性判断者。

**（4）样本量计算**

本研究采用PASS11.0软件进行样本量估算。根据预试验及文献研究结果得知，术后疲劳评分改变值对照组为-2.80±12.82，穴位按摩组为-33.63±10.01，放松训练组为-12.95±8.41，联合组为-40.90±7.53；医院焦虑抑郁量表中抑郁亚量表评分改变值对照组为12.50±3.41，穴位按摩组为10.67±3.49，放松训练组为11.15±3.27，联合组为11.03±3.19；医院焦虑抑郁量表中焦虑亚量表评分改变值对照组为11.38±3.74，穴位按摩组为11.00±3.40，放松训练组为10.18±3.22，联合组为10.45±3.21；在α=0.05，检验效能1-β=0.8的条件下，计算得每组样本量各37例，考虑失访、脱落等因素的存在，进行样本量20%的扩大，最终确定每组样本各45例，共计180例。完全随机设计多个样本均数比较的样本量估算公式如下：


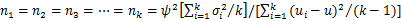


（其中，k为组数，*ψ*查统计用表中*ψ*值表获得）

**（5）取样及分组**

**①取样：**根据首次就诊的先后顺序，严格按照纳入排除标准选择受试对象，例数达到样本量即可。

**②分组：**按随机对照原则，采用SPSS19.0软件生成180个随机数，对这180个随机数编秩次为1-180号，研究对象按纳入先后抽取随机数，抽得随机数所对应秩次为1-45号、46-90号、91-135号和136-180号的研究对象分别归于对照组、实验1组、实验2组和实验3组。

**2、干预方法**

本研究采用随机对照实验的临床研究方法。研究对象按照随机分配原则，分组如下（4组）：

对照组：围手术期常规护理；

实验1组：围手术期常规护理+穴位按摩；

实验2组：围手术期常规护理+放松训练；

实验3组：围手术期常规护理+穴位按摩+放松训练。

**3、干预步骤**

**穴位按摩：**首先与患者做好沟通，解释穴位按摩的目的、程序和要求，以取得患者的理解和配合。患者取坐位，要求在安静、光线柔和的环境下进行，操作者将点法、按法、揉法施于百会、风池（双侧）、内关（双侧）、神门（双侧）、足三里（双侧）、三阴交（双侧）、承山（双侧）、昆仑（双侧）等穴位，先点压1分钟，再进行揉按，双侧穴位（风池、太阳、内关、神门、足三里、三阴交、承山、昆仑）两手同时揉按，每穴按摩时间为3-4分钟，100-120次/分钟，要求逐渐用力，由轻至重，由重至轻，反复按摩，以患者出现明显的酸、胀、热感为度，每日1次，每次约30min。

**放松训练：**首先与患者做好沟通，解释放松训练的目的、程序和要求，以取得患者的理解和配合。根据心理学专家Benson编制的渐进式放松训练而制成的中华医学会音像出版社出版的《自我放松教程》对患者进行全身放松训练。具体内容是保持环境安静，让患者取半靠位或卧位，使其感到舒适，集中精力，注意配合。让患者根据《自我放松教程》的提示音进行，让身体16组肌群(手和前臂、上臂、前额、面颊和鼻子、下领、颈部、胸肩、腹部、人腿、小腿、足)依次顺序绷紧、保持、放松。由研究者边示范边带患者做。每天1次，每次约20min。

干预从术后1天开始至术后5天结束，以上干预均由研究者本人操作。

**4、观测指标**

观察指标均由研究者本人评估记录，包括患者一般情况调查表、疲劳视觉模拟评分（Visual Analogue Scales，VAS）、围手术期疲劳测评量表（Identity-Consequence Fatigue Scale，ICFS）、医院焦虑抑郁量表（Hospital Anxiety and Depression Scale，HADS）等。

**表1 干预实施方案**

| **研究**  **对象** | **干预内容** | **评估内容** | | | | **评估人** |
| --- | --- | --- | --- | --- | --- | --- |
|  |  | **术前1天** | **术后1天** | **术后3天** | **术后5天** |  |
| 对照组 | 围手术期常规护理 | 一般情况调查表、VAS、ICFS、HADS评分 | VAS、ICFS、HADS评分 | VAS、ICFS、HADS评分 | VAS、ICFS、HADS评分 | 研究者 |
| 实验  1组 | 围手术期常规护理+穴位按摩 |  |  |  |  | 研究者 |
| 实验  2组 | 围手术期常规护理+放松训练 |  |  |  |  | 研究者 |
| 实验  3组 | 围手术期常规护理+穴位按摩+放松训练 |  |  |  |  | 研究者 |

**5、数据分析**

统计软件采用SPSS22.0。计量资料用均数、标准差描述，计数资料用频数、百分率描述；根据研究资料特征进行t检验或*x^2^*检验来比较研究对象一般资料和干预前VAS、ICFS、HADS、评分的基线情况，以评价四组基线的均衡性。分别对四组研究对象术后VAS、ICFS、HADS、评分得分进行的2×2析因方法分析单独效应、主效应和交互效应，再对四组研究对象进行完全随机设计资料4个时间点的重复测量方差分析，分别是术后1天、术后3天、术后5天和术后7天；四组研究对象干预前后的组内比较采用配对t检验（统计量为t），对于满足正态分布而方差不齐性的计量资料采用近似t检验（统计量为t′）；a值取0.05，P值均为双侧概率。

**6、年度研究计划及预期进展**

2019年05月～2019年10月：临床干预研究；

2019年11月～2019年12月：资料收集整理，进行统计分析；

2020年01月～2020年03月：撰写修改论文，完成论文终稿；

2020年04月～2020年05月：完成论文答辩。

**7、拟解决的关键问题**

①干预方法的科学性。

②样本的纳入与排除。

③干预方案的可行性。

④统计方法的准确性。
